# Latent classes of resilience in a nationwide sample of US adults during COVID-19 pandemic

**DOI:** 10.3389/fpsyg.2025.1462386

**Published:** 2025-08-05

**Authors:** Jing Huang, Joyline Chepkorir, Alden L. Gross, Phillip Phan, Noelle V. Pavlovic, Melissa D. Hladek

**Affiliations:** ^1^School of Nursing, Johns Hopkins University, Baltimore, MD, United States; ^2^Department of Epidemiology, Johns Hopkins Bloomberg School of Public Health, Baltimore, MD, United States; ^3^Carey Business School, Johns Hopkins University, Baltimore, MD, United States; ^4^Department of Medicine, Johns Hopkins Medicine, Baltimore, MD, United States; ^5^Center on Aging and Health, Johns Hopkins University, Baltimore, MD, United States

**Keywords:** resilience, COVID-19 pandemic, latent class analysis, adults, United States

## Abstract

**Background:**

The COVID-19 pandemic represented a unique and widespread challenge that profoundly impacted the well-being of individuals across all age groups. This study aimed to identify the latent classes of resilience among a nationwide sample of US adults and characterize these classes according to their socio-demographic profiles.

**Methods:**

We conducted a survey of 3,340 US adults from March to June 2020 through Qualtrics panel participants, stratified demographically to represent the US population by gender, race, age, and geographic region. A latent class analysis was performed to identify distinct profiles of resilience based on emotions, behaviors, physical symptoms, coping resources, and social support.

**Results:**

Four latent classes of resilience were identified among US adults during the COVID-19 pandemic: Low Mental/Physical Resilience (5.6%), Low Mental/Social Resilience (12.9%), Low Social Resilience (24.9%), and High Resilience (56.5%). The Low Mental/Physical Resilience class, which faced the highest mental risk, was notably distinguished by its members being younger, essential workers, and having children at home. Socially vulnerable groups, such as females and those with lower income, were more likely to be part of the Low Mental/Social Resilience and Low Social Resilience classes.

**Conclusion:**

Different groups of US adults may exhibit varying profiles of physical, mental, and social resilience during crises like the COVID-19 pandemic. The findings may help inform policies and interventions for mental health in future global health crises like COVID-19. During such crises, mental health support should be prioritized to essential workers and socially vulnerable groups, while accessible childcare services may particularly benefit parents who work.

## Introduction

The COVID-19 pandemic was a unique and widespread challenge that profoundly impacted the mental well-being of individuals across the globe, spanning all age groups. As isolation and quarantine measures (lockdown) were implemented to slow down the spread of virus and relieve healthcare burdens, people’s daily lives and established routines were extraordinarily disrupted. Many were faced with new challenges like home confinement, reduced social activities, and uncertain access to essential services, especially food and healthcare (Diotaiuti et al., 2023). As a result, people suffered from increasing negative emotions such as fear, worry, and anger (Fofana et al., 2020; Rothe et al., 2021; Richardson et al., 2022). A survey of United States (US) adults found that the prevalence of serious psychological distress increased from 3.9% in 2018 to 13.6% in April 2020 (McGinty et al., 2020). A meta-analysis on the prevalence of anxiety and depressive disorders during 2020 estimated that the pandemic caused an additional 76 million cases of anxiety disorders and 53 million cases of depressive disorders globally in 2020 (Santomauro et al., 2021).

Individuals reported highly variable emotional and mental experiences, despite the pandemic influencing the entire population (Richardson et al., 2022). Part of this can be attributed to the diversity of resilience across individuals. The American Psychological Association (n.d.) defined resilience as “the process and outcome of successfully adapting to difficult or challenging life experiences, particularly through mental, emotional, and behavioral flexibility and adjustment to external and internal pressures.” Resilience plays a crucial role in individuals’ ability to adapt to changes and navigate challenging circumstances, and hence is a protective factor in coping with the pandemic, for which people can “bounce back” in the face of adversity (Windle, 2011). People with higher resilience tend to recover more quickly after being exposed to stressors (Montpetit et al., 2010). Prior research has also demonstrated the protective role of resilience during large-scale disasters and traumatic events. For instance, studies following the 9/11 terrorist attacks in New York and 1999 floods in Mexico found that individuals with higher levels of resilience experienced lower rates of post-traumatic stress and depression, and recovered more quickly from the psychological impacts of these crises (Norris et al., 2009; Bonanno et al., 2007). In the context of the COVID-19 pandemic, one study found that higher levels of resilience were associated with more adaptive coping behaviors and less unhealthy behaviors (Vannini et al., 2021).

Resilience can be conceptualized as a multi-dimensional and ecological construct such that one person can have multiple resiliencies in different domains of their life. Additionally, a person is nested in family and community level resiliencies (or not), making a person’s environment an important contextual factor in their individual resilience capabilities (Hilliard et al., 2015; Southwick et al., 2014). As such, the degree of resilience depends on factors such as time, age, gender, specific challenges (e.g., COVID-19), available resources, and interactions with other people and environments (Southwick et al., 2014).

Previous studies examining resilience during the pandemic have largely applied a variable-centered approach (e.g., correlation, regression), which uses the score of a scale to indicate the level of resilience (Vannini et al., 2021; Killgore et al., 2020). While this approach is straightforward to implement and provides valuable information, it does not capture the variation in patterns and dimensions of resilience that we know exist in the population. A total resilience score is inadequate for distinguishing between subgroups with varying resilience characteristics, as individuals with identical total scores may still exhibit varied resilience along different dimensions. Consequently, a single score cannot provide sufficient information to support specific interventions for the subgroups of people with different resilience characteristics.

To close this knowledge gap, latent class analysis (LCA), a person-centered approach, is used to identify the resilience phenotypes among the population. LCA is a statistical method that recognizes latent classes may be useful for classifying subgroups into mutually exclusive categories based on their homogeneity across a set of observed characteristics (Lanza and Rhoades, 2013). A growing number of studies have used person-centered approaches to identify the latent classes of resilience in different contexts and populations (Janousch et al., 2022; Lines et al., 2020; Luo et al., 2022). However, to our knowledge, no study has investigated latent classes of resilience in the general population of US adults during the pandemic. Previous studies applying person-centered approaches have primarily focused on emotional experiences related to COVID-19, without capturing the profiles of multidimensional resilience. For instance, one study identified four latent classes of negative and positive emotional responses during the COVID-19 pandemic (Richardson et al., 2022). There remains a need for including more comprehensive assessments of mental experiences to better capture the multidimensional nature of resilience. Moreover, resilience research during COVID-19 has often centered on specific populations, such as young adults and pregnant women (Werchan et al., 2022; Schmidt et al., 2022). The COVID-19 pandemic, as a universal stressor, provided a unique opportunity to enhance our understanding of resilience in a general adult population. Therefore, the aims of this study were to (1) identify the latent classes of resilience amongst a nationwide sample of US adults; (2) characterize and explore the resilience classes according to their socio-demographic profiles.

### Conceptual framework

This study is grounded in the Multi-System Model of Resilience developed by Liu and colleagues (Liu et al., 2017), which conceptualizes resilience as a dynamic, multidimensional process involving multiple systems: within individuals, between individuals, and at the broader socio-political level. The model organizes resilience into three concentric layers: core resilience, internal resilience, and external resilience. Core resilience encompasses factors intrinsic to individuals, such as physiological responses to adversity, health behaviors, and biological indicators. Internal resilience includes elements that develop over time through interpersonal sources, such as family, friends, and personal experiences, and may involve emotional regulation, psychological strength, coping strategies, and social resources. External resilience refers to the broader socio-ecological context, such as socioeconomic status and access to social services.

Guided by this framework, we incorporated core and internal resilience factors into the person-centered LCA, focusing on physical symptoms, health behaviors, emotional indicators (e.g., anxiety, depression, loneliness, and trauma), coping resources, and social support. External resilience factors were examined as sociodemographic characteristics across the latent classes.

## Materials and methods

### Setting, data collection, and sample

Data were collected through a survey of 500,000 Qualtrics panel pre-consented participants, stratified to represent the US population by gender, race, age, and geographic region. The survey instrument consisted of validated self-report scales measuring mental states, social networks, and behaviors, in addition to demographic information. Participants were paid a $5.15 incentive to participate in the survey. Prior research has shown that monetary incentives can effectively increase response rates without significantly affecting sample composition or response distributions (Singer and Ye, 2013). Data quality (i.e., manipulation checks for respondent fatigue, respondent inattention, social desirability, etc.) was checked at the point of collection. The survey was conducted between March to June 2020, shortly after the World Health Organization declared COVID-19 a global pandemic and as lockdowns, travel restrictions, and school closures were widely implemented across the US. A total of 3,340 individuals responded to the survey. The response data were checked against the general population statistics to ensure representativeness.

### Measures

Latent classes of resilience were identified based on participants’ responses on questionnaires assessing their emotions, behaviors, physical symptoms, coping resources, and social support during COVID-19 pandemic.

Emotional indicators included depression, anxiety, loneliness, and trauma. Depression was measured by the Patient Health Questionnaire-2 (PHQ-2), with a total score ranging from 0 to 6 and a score ≥3 was categorized as depressed (Kroenke et al., 2003). A previous study suggested that a PHQ-2 score ≥3 had a sensitivity of 83% and a specificity of 92% for major depression, using interviews by mental health professionals as the reference, and its diagnostic accuracy was comparable to that of the PHQ-9 (Kroenke et al., 2003). Anxiety was measured using seven items from the Generalized Anxiety Disorder-7 scale (GAD-7) with a cutoff of 10 (Spitzer et al., 2006). Loneliness was measured by the three item UCLA Loneliness Scale (Hughes et al., 2004). The self-report total score can range from 3 to 9 with a score ≥6 considered as lonely (Steptoe et al., 2013). “Trauma Symptoms” was assessed using nine items drawn from the Severity of Posttraumatic Stress Symptoms for Adults, National Stressful Events Survey PTSD Short Scale (NSESSS) (Kilpatrick et al., 2013). We categorized participants that reported *any* traumatic symptoms as traumatized (NESSS score ≥0), since most of the participants did not report any symptoms.

Behavioral indicators consist of alcohol use, drug use, and food addiction. Alcohol Use was measured by three items from the Alcohol Use Disorder Identification Test (AUDIT-C) (Dawson et al., 2005; Bush et al., 1998), a screening tool that identifies individuals who are hazardous drinkers or have active alcohol use disorders including alcohol overuse or dependence. The scale evaluates the frequency of alcohol consumption, the numbers of drinks per day, and the frequency of having six or more drinks on one occasion. A score of ≥ 4 for men and ≥ 3 for women, based on a total score range of 0–12, was used to determine the presence of alcohol use disorder. Drug use was measured with the NIDA-Modified ASSIST Level 2-Substance Use for Adult questionnaire, which asked about the frequency of painkiller, stimulant, and sedative use in the past 2 weeks (WHO ASSIST Working Group, 2002). Individuals were categorized as overusing drugs if they reported having used any of these types of drugs for more than half the days or nearly every day. Food addiction was determined when an individual had two or more symptoms plus impairment or distress based on the nine items of the Modified Yale Food Addiction Scale (Schulte and Gearhardt, 2017).

Physical indicators consisted of perceived cognitive deficit and pain. Perceived cognitive deficit was assessed by the 5-item Perceived Deficits Questionnaire—Depression (PDQ-D-5) (Cha, 2016), which is a measure of self-reported cognitive impairment related to depression with a score ≥9 as cognitively deficient. Pain was indicated by a self-report dichotomous measure on whether they had pain on most days.

Coping resources were evaluated with the Coping Resources Inventory (Matheny et al., 1993), which assesses an individual’s perceived coping resources in response to stress. The total scores ranged from 0 to 120 with a score between 0 and 73 regarded as having low coping resources. Social support was indicated by the social networks of both family and friends. Family network and friendship networks were each assessed from two subscales of the Lubben Social Network Scale, which measured the numbers of family members or friends with whom the respondent interacted, felt close to, and felt at ease (Lubben et al., 2006). The total scores range from 0 to 30 and we categorized the scores between 0 and 7 as low social support.

In addition, the respondents’ sociodemographic characteristics were collected via self-report, namely age, sex, race, education, marital status, household income, whether had children at home, whether an essential worker, and working hours per week.

### Statistical analysis

An LCA was conducted to identify the latent classes of resilience during the COVID-19 pandemic based on the responses of pain, trauma, depression, anxiety, loneliness, coping, social network, perceived cognitive deficit, and food, alcohol, and drug use. Models with different numbers of classes were performed, starting from 2 to 6 classes. The optimal number of classes for the model was determined by the Akaike information criterion (AIC), Bayesian information criterion (BIC), the Lo-Mendel-Rubin likelihood ratio test (LMRT), the bootstrap likelihood ratio test (BLRT), and entropy (Weller et al., 2020). Lower values of AIC and BIC indicated a better fit. LMRT and BLRT are tests for comparing models with *k* classes and *k*-1 classes. A *k* class model is better than the *k*-1 class model if a significant *p*-value (*p* < 0.05) is found, otherwise, the *k*-1 class model provides a more parsimonious fit to the data. An entropy value closer to 1 is preferred, but ≥ 0.70 is acceptable (Celeux and Soromenho, 1996). Among these criteria, BIC and LMRT have been shown to perform best in determining the optimal number of classes in latent class analysis (Nylund et al., 2007). After determining the optimal number of classes, we examined social and demographic characteristics using post-estimation statistics within the model. The descriptive statistics were performed on Stata 18.0 and the LCA was conducted with Mplus 7.4.

## Results

### Sample characteristics

The total study sample consisted of 3,340 participants, 75% (*n* = 2,507) were less than 65 years old and 25% (*n* = 833) were 65 years or older. About half of the participants (49.9%) were female (*n* = 1,658). Most study participants were white (76.8%), and the rest self-identified as Asian/Pacific Islander (9.6%), Hispanic (6.6%), Black (5.6%), or Other (1.4%). Most study participants were married or cohabiting (64.4%) and obtained some college or higher education (83.7%), while 48.8% of them had $81,000 and higher annual household income. In terms of work status, 35.7% of respondents were essential workers and 36.5% worked 40 h or more per week. Additionally, 38.0% of respondents had children at home and 16.7% of them had children at home while being an essential worker.

### Latent classes of resilience

The model fit statistics with different numbers of classes are displayed in Table 1. The 4-class model was selected because it reported marked improvements in AIC and BIC and obtained significant *p*-values for LMRT and BLRT when compared to the 3-class model. Meanwhile, when comparing 5-class model to the 4-class model, the AIC and BIC value did not show improvement and LMRT was not significant, which indicated that the 5-class model was not better than the 4-class model. The entropy of the 4-class model was higher than 3-class and 5-class models and in the acceptable range for class distinction.

**Table 1:** Model fit statistics.

Classes	AIC	BIC	LMRT *p* value	BLRT *p* value	Entropy
2	31858.382	31998.998	<0.001	<0.001	0.910
3	31342.497	31556.477	<0.001	<0.001	0.756
4 *	30988.844	31276.189	<0.001	<0.001	0.791
5	30904.248	31264.958	0.0998	<0.001	0.777
6	30842.483	31276.557	0.2413	<0.001	0.783

BIC, Bayesian Information Criterion; AIC, Akaike Information Criterion; LMRT, Lo–Mendell–Rubin Test; BLRT, Bootstrapped Likelihood Ratio Test; *Selected model.

**Figure 1:**
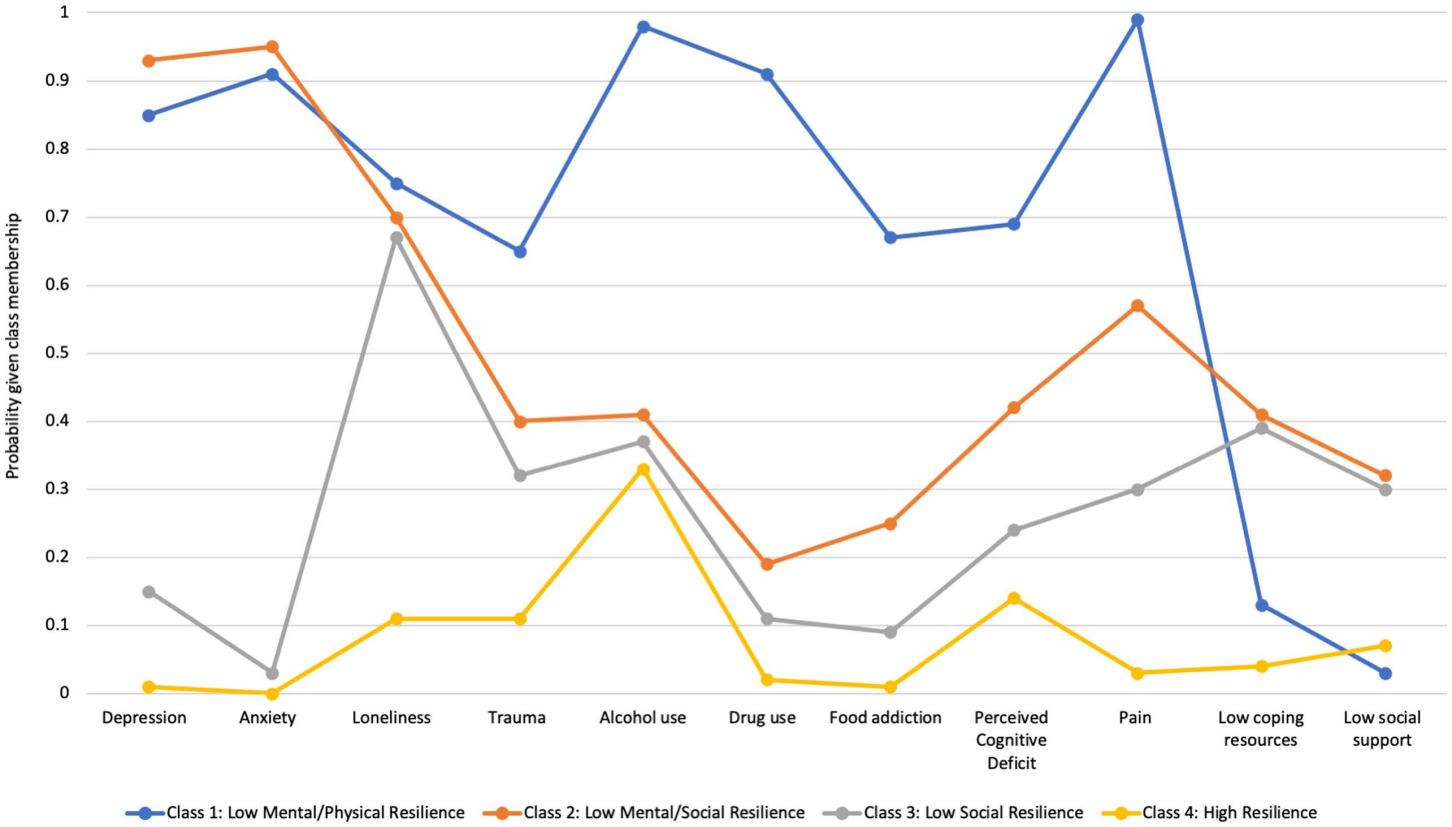
Class probabilities.

As shown in Figure 1, Class 1—Low Physical/Mental Resilience (5.5%) exhibited prominently high probabilities for all the items, except for coping resources and low social networks. They were highly likely to report physical symptoms, negative emotions, and unhealthy behaviors, despite good coping resources and social networks. Class 2—Low Mental/Social Resilience (14.1%) had higher mood-associated symptoms with the highest probabilities of having depression and anxiety. They also had the highest likelihood of having low coping resources and social networks. Class 3—Low Social Resilience (24.3%) showed similar probabilities of having low coping resources, limited social networks, and experiencing loneliness as Class 2, but had much lower risks of depression and anxiety compared to Classes 1 and 2. Class 4—High Resilience (56.0%) showed the lowest probabilities across all the classification items. They had a low risk of suffering from pain, cognitive deficits, and mood issues. Also, they had sufficient social support and few unhealthy behaviors.

As reported in Table 2, when examining the characteristics of the identified classes, Class 4 was significantly older than the other classes. While the mean ages of the other classes ranged from 33 to 41 years, the mean age of Class 4 was 54 years. Class 2 and Class 3 had significantly more females than Class 1 and Class 4. Compared to Class 4, the other three classes were significantly less likely to be White but more likely to have high school or lower education (more detailed race and education information is available in Supplemental Table 1). Classes 2 and 3 were significantly less likely to be married and have an annual household income greater than $81,000, compared to Classes 1 and 4. In terms of income, Class 1 was significantly more likely to be essential workers, having children at home, or both, in comparison to Class 4.

**Table 2:** The characteristics of the total sample and each class.

Characteristic	Total (*N* = 3,340)	Class 1 – Low Mental/Physical Resilience (5.5%)	Class 2 – Low Mental/Social Resilience (14.1%)	Class 3 – Low Social Resilience (24.3%)	Class 4 – High Resilience (56.0%)
	n (%)	%	%	%	% (Ref)
Age (Mean ± SD)	47.3 ± 17.7	33.02 ± 0.68*	38.28 ± 0.73*	40.62 ± 0.77*	53.91 ± 0.45
Female (ref = male)	1,658 (49.9%)	42.8%	64.5% *	56.9% *	44.0%
White (ref = non-white) ^a^	2,565 (76.8%)	69.4% *	73.4% *	66.7%*	82.8%
High school education or less (ref = some college or higher)	546 (16.3%)	17.8%	26.7% *	19.7% *	12.1%
Married/Cohabiting (ref = unmarried)	2,151 (64.4%)	74.6%	50.4% *	46.5% *	74.7%
Annual household income ≥$81,000 (ref = less than $81,000)	1,222 (48.8%)	64.4%	33.8%*	34.5% *	57.6%
Working 40 + hours/week (ref = < 40 h/week)	1,218 (36.5%)	43.7%	35.7%	36.0%	36.2%
Essential worker	1,193 (35.7%)	66.4% *	32.5%	34.6%	34.2%
Children at home	1,269 (38.0%)	82.0%*	42.5%*	37.2%	32.8%
Essential worker + Children at home	559 (16.7%)	55.4%*	15.7%	15.6%	13.7%

ref, reference group, *p-value ≤ 0.05 in comparison to class 4.

## Discussion

This study is among the first to identify the latent classes of resilience in a nationwide sample of US adults during the COVID-19 lockdown. We identified four unique and conceptually meaningful latent classes of resilience, including Class 1—Low Mental/Physical Resilience (5.5%), Class 2—Low Mental/Social Resilience (14.1%), Class 3—Low Social Resilience (24.3%), and Class 4—High Resilience (56.0%). These classes were derived from distinct patterns across 11 resilience-related indicators—including emotional symptoms, behavioral health risks, cognitive and physical symptoms, and protective resources—guided by the Multi-System Model of Resilience framework. They also displayed differed by sociodemographic characteristics, including age, gender, race, education, marital status, income, being an essential worker, and having children at home.

A notable finding is that more than half of the adults demonstrated high resilience during COVID-19 lockdown (Class 4). While they occasionally consumed alcohol, overall, they adapted well to the situation. In contrast, the remaining classes endured varying degrees of challenges during the lockdown. Class 1—Low Mental/Physical Resilience, representing a small proportion of the population, appeared to suffer the most during the pandemic. They had the most physical and mental symptoms along with the highest unhealthy behaviors including alcohol use, drug use, and food addiction. This group had the highest likelihoods of being younger, having children at home, and being essential workers during the pandemic. Class 2—Low Mental/Social Resilience – although reported fewer physical symptoms than Class 1, uniformly suffered from anxiety and depression symptoms. They also had the lowest coping resources and social support, suggesting increased vulnerability to decline over time. This class included more females and unmarried persons with lower educations and incomes. Class 3—Low Social Resilience showed good adaptation with fewer symptoms and unhealthy behaviors compared to Classes 1 and 2. However, Class 3 individuals also experienced loneliness and had relatively lower coping resources and social support, which suggest potential vulnerabilities that require additional attention. In terms of demographics, Class 3 individuals tended to be females, identify as non-White, unmarried, and with lower incomes.

Our findings support the notion that impact of the COVID-19 pandemic varied across different demographic groups. Younger individuals, females, non-White individuals, and those with lower income were more likely to exhibit lower resilience. This is consistent with the findings from the previous studies that these factors were associated with higher risks for psychological stress and mental health concerns related to COVID-19 (Monnig et al., 2023; Liu et al., 2020; Shuster et al., 2021; Kujawa et al., 2020). Prior research has suggested that the COVID-19 pandemic significantly widened mental health disparities in many countries, primarily stemming from underlying structural inequities (Purtle, 2020; Lee et al., 2023). Individuals facing structural vulnerabilities were disproportionately prone to experiencing more severe health impacts of the pandemic for several reasons (World Health Organization, 2020). They might be more likely to be predisposed to pre-existing mental health conditions, encountered limitations or interruptions in accessing mental health services, and grappled with additional challenges such as unemployment, social isolation, domestic violence, and loss of loved ones (Purtle, 2020; World Health Organization, 2020). This study adds a resilience perspective to these findings. Older age was associated with better resilience, likely due to accumulated life experiences of adversity and improving coping skills over time as people age (Wister et al., 2022). Conversely, socially vulnerable groups—such as females, non-White individuals, and those with lower incomes—may experience unique cumulative stress burdens compounded by this pandemic and structural inequities leading to a mismatch in stress burden and resilience capacity (Liu et al., 2017).

Furthermore, when looking into the characteristics of the most at-risk class, Class 1—Low Mental/Physical Resilience, we found females and lower income were not a significant factor but having children at home, being an essential worker, and working over 40-h per week were risk factors. These individuals shouldered the most responsibilities among the population we surveyed, and yet lacked adequate support, resulting in the lowest resilience levels observed. Specifically, four in five had children at home and over half were essential workers, a large difference compared to the other 3 classes. As schools and daycare centers closed from COVID-19-related movement restrictions, responsibilities for childcare largely fell on the female caregivers. Concomitantly, parents working from home did not mean reduced workload but rather increased workload due to an additional 8 h of child rearing when the latter would have been in school (UNICEF Innocenti 2020). Meanwhile, social distancing mandates limit the amount of informal care provided by friends or extended family members, such as grandparents (Linnavalli and Kalland, 2021). Grandparental care is a common option for covering childcare responsibilities for working parents but during the pandemic, this was not available as older adults were asked to be more sensitive to the risks of contracting the disease (Blau, 2001). Our findings are consistent to previous studies which found that the mental health and well-being of parents of families with children declined during the COVID-19 lockdown (Gassman-Pines et al., 2020; Brown et al., 2020). It is critical for clinicians to assess and address the emotional and mental health of individuals during such public health emergencies and for stakeholders to connect individuals to relevant resources.

For essential workers, there were additional challenges besides the increased stress of childcare and household responsibilities. These individuals faced more demanding workloads, risked losing their incomes, and their own health (Harry et al., 2022; Warren and Lyonette, 2021). They had trouble in securing grandparental childcare because of the concerns over the severity of the disease for older adults. Our findings echo previous findings that essential workers experienced higher mental health burden during the pandemic, and experienced mental health concerns such as anxiety, depression, and even suicide ideation (Monnig et al., 2023; Fang et al., 2020; Sugg et al., 2021).

Our study uniquely examined the proportion of individuals juggling both parenting and essential worker responsibilities across the latent classes. The Class 1 essential worker parents may have exceptional abilities around resilience but, due to the volume and cumulative burden of external stressors added to their system, their resilience capabilities were taxed beyond the coping resources they had available. It is an incomplete statement to say this class was simply “non-resilient.” These findings highlight the importance of providing mental health support and childcare for essential workers during crises. These findings also speak to the importance of including structural supports in conversations about individual level resilience.

This study has some notable strengths. First, the study sample was a large nationwide sample, which increases the generalizability of our findings. Second, the study utilized well recognized validated and reliable instruments to assess the emotional, behavioral and mental status of participants, which gives us confidence in the results we found. However, it is also important to acknowledge some limitations. First, the study sample mainly consisted of White adults (76.8%), which might limit the generalizability of the study findings. Second, the findings are based on cross-sectional data collected during the COVID-19 lockdown in June 2020 and causality cannot be assumed. It is possible that resilience during the COVID-19 pandemic evolved over time, especially as more public health information and vaccines became available. Therefore, future research employing a longitudinal design is recommended to track the development of resilience profiles over time. Third, this study utilized brief assessment tools, such as PHQ-2. While they are validated and widely used, they may not capture the full spectrum of mental health outcomes experienced during the COVID-19 pandemic. Future research may consider utilizing more extensive mental health measures to gain more comprehensive insights into the varied mental health experiences of individuals during crises like the COVID-19 pandemic.

The study findings fostered a better understanding of resilience at a population level, with significant practical implications. Although we cannot conclusively attribute our findings to the COVID-19 pandemic due to the absence of a matched pre-pandemic comparison group, it is crucial to recognize that the observed resilience phenotypes may extend beyond this specific health crisis. Instead, they may reflect broader patterns of adaptation employed in response to various stressful life events. Future research may consider developing more targeted interventions to improve resilience based on these identified profiles.

A growing body of research (Liu et al., 2017; Kaye-Kauderer et al., 2021) no longer approaches resilience as a fixed individual trait but as a dynamic, interactive process that can be developed and strengthened over time. As informed by the Multi-System Model of Resilience, resilience can be enhanced at multiple levels: within-individual, interpersonal, and socio-ecological. At the within-individual level, our findings highlight the importance of physical health management, such as pain control, and interventions to reduce unhealthy behaviors while promoting healthy behaviors, particularly for individuals in Class 1 (Low Mental/Physical Resilience). During crises like the pandemic, when healthy behaviors like physical exercise may be restricted, it is crucial to provide timely, adaptive guidance on maintaining health (D'Oliveira et al., 2022).

On the interpersonal level, numerous strategies can support resilience enhancement. Psychoeducation programs that provide training on emotional regulation, active coping, help-seeking, and cognitive reappraisal can empower individuals facing adversity (Kaye-Kauderer et al., 2021), especially those in Classes 1 and 2, who reported high levels of emotional distress and traumatic symptoms. Expanding interpersonal resources and strengthening support networks are also critical, especially for individuals with low social resilience in Classes 2 and 3. Digital tools—such as social media, video conferencing, and online social activities—can help maintain connectedness during crises like COVID-19 (Shah et al., 2020). However, it is essential to ensure equitable access to these technologies and to protect user safety (Shah et al., 2020).

At the broader socio-ecological level, our findings also offer important insights to inform policies and social services aimed at resilience. We found that non-white races, lower socioeconomic status, parenting responsibilities, and essential worker roles were associated with suboptimal resilience profiles. Consistent with previous research (Richardson et al., 2022; Lopez et al., 2021), we urge policymakers to address structural inequities that impact racial/ethnic minorities, low-income households, and rural communities. Implementing protective policies—such as those that ensure job security, reduce pay inequities, provide affordable housing, and offer unemployment support—can help alleviate economic stress within these groups. Extending healthcare access to socially vulnerable populations and frontline essential workers is also crucial to ensure they receive necessary counseling and mental health services. Additionally, a pressing need exists for more accessible and affordable childcare services for essential workers, highlighting the importance of supporting those on the front lines and their families in times of crisis.

## Conclusion

In conclusion, this study identified four latent classes of resilience among US adults in the context of the COVID-19 pandemic. The latent classes exhibit distinct multidimensional resilience profiles and sociodemographic characteristics. The findings have significant implications at multiple levels for public health policy and intervention strategies aimed at enhancing mental health in future global health crises like COVID-19. We recommend prioritizing mental health support for essential workers and socially vulnerable groups, who were more likely to exhibit lower resilience during the crisis and may face persistent barriers to accessing care. Additionally, expanding access to affordable childcare services may be a critical strategy to support working parents and reduce stress during times of crisis. Implementing such targeted policies and interventions can help strengthen population resilience and better prepare communities for future public health emergencies.

## Data Availability

The original contributions presented in the study are included in the article/Supplementary materials, further inquiries can be directed to the corresponding author.
