# Supplementary material for: Latent classes of resilience in a nationwide sample of US adults during COVID-19 pandemic
Source: Front Psychol. 2025 Aug 5;16:1462386. doi: 10.3389/fpsyg.2025.1462386 (PMC12361235; doi:10.3389/fpsyg.2025.1462386)
Supplement: Supplementary file 1 [file Table_1.docx]

Supplemental Table 1. The characteristics of the total sample and each class (detailed)

|  | Total  (N=3,340) | Class 1 – Low Mental/Physical Resilience (5.5%) | Class 2 – Low Mental/Social Resilience (14.1%) | Class 3 – Low Social Resilience (24.3%) | Class 4 – High Resilience (56.0%) |
| --- | --- | --- | --- | --- | --- |
| Characteristic | n (%) | % | % | % | % (Ref) |
| Age (Mean ± SD) | 47.3 ± 17.7 | 33.02 ± 0.68* | 38.28 ± 0.73* | 40.62 ± 0.77* | 53.91 ± 0.45 |
| Female (ref = male) | 1,658 (49.9%) | 42.8% | 64.5% * | 56.9% * | 44.0% |
| Race |  |  |  |  |  |
| White | 2565 (76.8%) | 69.4% * | 73.4% * | 66.7%* | 82.8% |
| African American | 186 (5.6%) | 11.3%* | 8.2%* | 6.9%* | 3.7% |
| Asian American | 319 (9.6%) | 7.5% | 8.5% | 15.4%* | 7.5% |
| Hispanic | 221 (6.6%) | 11.9%* | 8.6%* | 9.2%* | 4.5% |
| Education |  |  |  |  |  |
| High school education or less | 546 (16.3%) | 17.8% | 26.7% * | 19.7% * | 12.1% |
| 1-2 years of college | 663 (19.9%) | 15.6% | 23.7%* | 22.5% | 18.2% |
| 3-4 years of college | 1,214 (36.4%) | 37.6% | 30%* | 36.2% | 37.9% |
| Postgraduate | 917 (27.5%) | 29.0% | 19.6%* | 21.6%* | 31.8% |
| Married/Cohabiting  (ref = unmarried) | 2,151 (64.4%) | 74.6% | 50.4% * | 46.5% * | 74.7% |
| Annual household income ≥$81,000 (ref = less than $81,000) | 1,222 (48.8%) | 64.4% | 33.8%* | 34.5% * | 57.6% |
| Working 40+ hours/week  (ref = < 40 hours/week) | 1218 (36.5%) | 43.7% | 35.7% | 36.0% | 36.2% |
| Essential worker | 1,193 (35.7%) | 66.4% * | 32.5% | 34.6% | 34.2% |
| Children at home | 1269 (38.0%) | 82.0%* | 42.5%* | 37.2% | 32.8% |
| Essential worker + Children at home | 559 (16.7%) | 55.4%* | 15.7% | 15.6% | 13.7% |
| Notes: ref = reference group, *p-value ≤ 0.05 in comparison to class 4 | | | | | |
